# Implementation of High Dose-rate Brachytherapy for Cervix Cancer in a Low-income Country

**DOI:** 10.29024/aogh.2377

**Published:** 2018-11-05

**Authors:** Sommer R. Nurkic, Ana Isabel Ocampo, Mario Josè Pinell Gadea, Julie Greenwalt, Mario Jose Vicente, Anielka Lucia Velasquez, Lisbeth Concepcion Lopez Peralta, Franck Soto Herrera, Osmara Calero Romero, Francisco Lopez Tenorio, Harving Lorente Zamora, Luis Matamoros Munguia, Anamaria Yeung

**Affiliations:** 1 Department of Radiation Oncology, University of Florida, Gainesville, FL, US; 2 El Centro Nacional de Radioterapia, Managua, Nicaragua, NI

## Abstract

**Background:**

The purpose of this study is to detail the implementation of HDR brachytherapy at the only radiotherapy center in Nicaragua.

**Methods:**

Patients are treated with external-beam radiotherapy to 46–50Gy at 2Gy/fraction to the pelvis. A gynecologic examination is performed weekly. Once the cervical os is visualized, brachytherapy is initiated. HDR is delivered in four fractions of 7Gy twice weekly. HDR occurs in two phases: preparation and delivery. Treatment preparation occurs in the procedure room, which includes anesthization, cervical dilation, and brachytherapy applicator placement using fixed-geometry tandem and ring with a rectal blade. The applicator is immobilized and the patient transferred to a stretcher and transported to the treatment delivery room. HDR is performed with the patient on the stretcher to minimize motion. AP and lateral films are taken using portable equipment. Physics staff digitize Point A, rectal point, and bladder point. A standard plan is loaded and approved prescribing 7Gy to Point A. If dose to the rectal or bladder points exceeds the constraint, the applicator is adjusted or vaginal packing is added and films repeated.

**Results:**

Nearly 10 years after implementing the HDR program, the center is treating 11–15 women with HDR brachytherapy for cervix cancer daily. Because the procedure is carried out over two separate rooms, patients can be staggered and more treated daily. The rooms turn over every 45 minutes.

**Conclusions:**

HDR brachytherapy for cervix cancer has been successfully established in Nicaragua. Significant challenges remain, and there is a role for developed countries to collaborate.

## Introduction

Nicaragua, a low- middle-income country (LMIC) situated in the isthmus of Central America, has one of the highest incidence and mortality rates related to cervical cancer in all of the Americas. It is estimated that the age-standardized incidence of newly diagnosed cervix cancer is 36.2 per 100,000 in Nicaragua, compared to 6.6 per 100,000 in the United States. Similarly, the mortality rate of cervical cancer is 18.3 per 100,000 in Nicaragua, compared to 2.7 per 100,000 in the United States [1]. Currently, a single radiation oncology center located in the capital of Managua services the entire country and adjacent region. In an effort to improve access to treatment and efficiency, Nicaragua – with the support of the International Atomic Energy Agency (IAEA) – initiated a high dose-rate (HDR) therapy program at El Centro Nacional de Radioterapia. Here we describe the process and procedure of implementing Nicaragua’s first and only HDR brachytherapy program for the treatment of cervical cancer.

## Background

El Centro Nacional de Radioterapia is staffed by two full-time radiation oncologists, seven radiation oncology residents, and two medical physicists. There are two cobalt-60 (^60^Co) treatment machines and one iridium-192 (192-Ir) HDR unit. Motivation to build a radiotherapy center began in the 1980s, starting with a ^60^Co machine and a low dose-rate (LDR) brachytherapy program. The HDR program was subsequently launched in 2007 to address the growing demand for curative treatment for their cervical cancer patients.

## Treatment Overview

Patients with early-stage and locally advanced cervical cancer are typically treated with primary chemoradiation. Chemotherapy includes weekly cisplatin prescribed to 40 mg/m^2^. Radiotherapy treatment is delivered both with a course of external-beam radiotherapy (EBRT) and HDR brachytherapy. The EBRT treatment involves a daily fraction of 2Gy to a total dose of 46– to 50Gy to the pelvis and regional lymph nodes; women presenting with parametrial wall involvement or pelvic sidewall invasion receive an 8– to 10Gy sidewall boost. A weekly gynecologic examination is performed to determine the appropriate time to initiate HDR brachytherapy, which is guided by visualization of the cervical os. HDR brachytherapy is delivered in four fractions of 7Gy delivered to Point A usually starting during the last two weeks of EBRT.

The HDR plan is prepared by the medical physicist and approved by the radiation oncologist. The doses to Point A, the bladder, and the rectum are reviewed. If the dose to the bladder or rectum is too high, they will adjust the applicator and repeat the films. If the dose to the bladder and rectum remain high after applicator adjustment, the dose prescribed to Point A will be lowered to meet the bladder and rectal dose constraint. HDR dose constraints to the bladder and rectal points are <80% and <60%, respectively [2]. The dose to Point A and bladder and rectal points are in congruence with those outlined by the International Commission on Radiation Units and Measurement [3].

## Program Workflow

**Figure 1:**
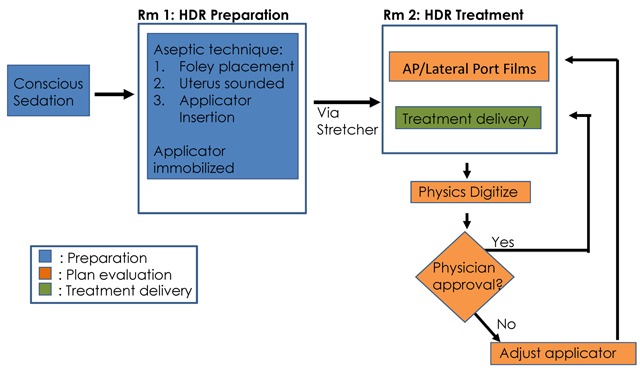
The treatment workflow implemented at Centro Nacional de Radioterapia from patient sedation to treatment delivery.

**Figure 2:**
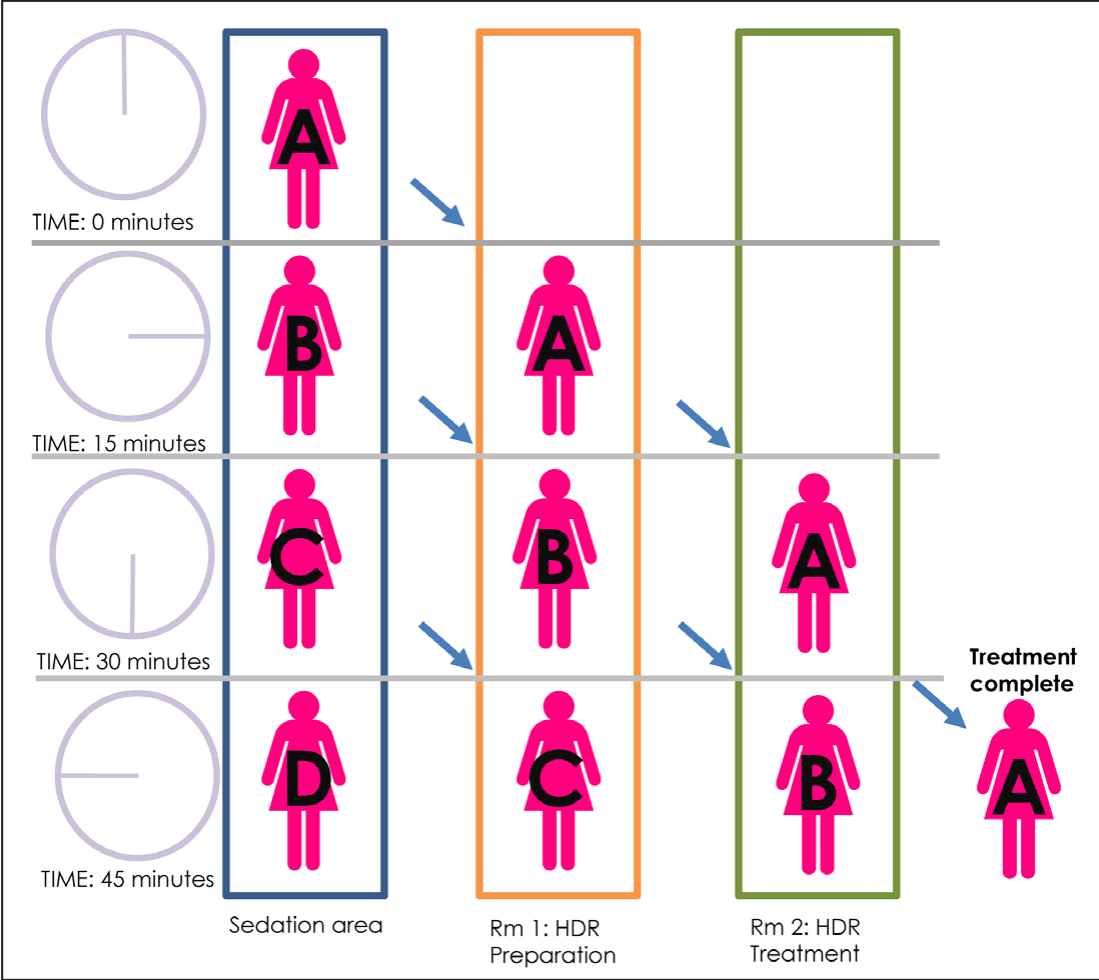
A schematic of patient flow through three areas: sedation area, HDR preparation room, and HDR treatment room. The total treatment time is usually 45 minutes, which can vary by source age.

To accommodate the high volume of cervical cancer patients requiring HDR brachytherapy at El Centro Nacional de Radioterapia, a workflow was created to keep women moving through various phases of HDR preparation and delivery (Figure 1). In general, treatment takes place in two separate phases: treatment preparation and treatment delivery—both of which occur in separate rooms to simultaneously advance multiple women through various phases of treatment (Figure 2).

Treatment preparation is completed in the procedure room, which is equipped with an examination table with stirrups. The patient is placed in the lithotomy position. A nurse anesthetist provides conscious sedation using a combination of midazolam, fentanyl, and propofol; the anesthetic used is largely guided by availability. The patient’s oxygen saturation and heart rate are monitored before, during, and after sedation delivery. An anesthesiologist is available on sight and signs off on the plan as administered. Using an aseptic technique, a Foley catheter is placed with 7 cm^3^ of contrast in the balloon; the uterus is sounded and dilated without image guidance; and after that, the brachytherapy applicators are placed in the uterus and vagina. Most cases are done using a 4 to 6 cm fixed-geometry tandem-and-ring applicator with a rectal blade. Vaginal packing is not typically used unless required to improve plan dosimetry. Immobilization of the applicators is achieved by taping gauze around the applicators and over the perineum. The patient is then transferred to a gurney and transported to a separate room to begin the HDR treatment delivery process.

HDR treatment delivery is performed with the patient on the same gurney to minimize motion. Daily imaging with anterior-posterior and lateral films are taken using portable x-ray equipment for planning and verification. Physics staff digitize Point A, the rectal point, and the bladder point. A standard plan is selected from a preloaded library of plans and 7Gy is prescribed to Point A; then the plan is approved by the physician. HDR dose constraints to the bladder and rectal points are <60% and <80% of the dose to Point A, respectively [2]. The dose to Point A and bladder and rectal points are in congruence with those outlined by the International Commission on Radiation Units and Measurement [3]. If the dose to the rectal or bladder point exceeds the constraint, the applicator is adjusted or vaginal packing is added and films are repeated. If the dose to the rectal or bladder point still exceeds the constraint, the dose to Point A is lowered.

With HDR treatment preparation and HDR treatment delivery carried out in separate rooms, the center is able to treat 11 to 15 women with HDR brachytherapy for cervix cancer daily. The exact number of women treated is dependent upon the age of the HDR source. In the absence of complication or delay, the procedure room and HDR treatment room are turned over every 45 minutes. Most importantly, the center has found that by staggering patients throughout different stages of treatment, more women may be treated daily.

## Program Implementation

The availability of HDR treatment for cervical cancer was dependent upon getting appropriate and adequate resources into Nicargaua. First, getting the HDR afterloader (microSelectron HDR – Genie Afterloading System, Nucletron, Columbia, MD) through customs required significant coordination between IAEA, customs officials, and the medical center. Once the afterloader was imported and installed, IAEA offered courses and instruction to physicians and medical physicists on treatment planning and delivery. Because of the center’s significant historical experience with LDR brachytherapy, the radiation oncologists were well-trained and familiar with an applicator-based procedure and recognition of proper geometry to reduce treatment toxicity. IAEA continues to offer ongoing support, educational materials, and instruction to physicians and medical physicists in the event of machine or software malfunction. Furthermore, IAEA is responsible for replacement and repair of both the Iridium and Cobalt sources, which are exchanged on an as-needed basis. This exchange is complex and requires collaboration from many, including the physicians and physicists at the center, IAEA, and the Nicaraguan government.

Allocation of a specific room for HDR treatment delivery further optimized the efficiency of the center to treat patients. Having an HDR-designated room apart from the ^60^Co vault was essential to providing simultaneous care for patients undergoing EBRT and HDR treatment as well as minimizing scheduling complications between HDR and EBRT treatments.

Lastly, use of fixed-geometry applicators with a preloaded library of standard plans streamlined the HDR treatment delivery. Fixed-geometry applicators simplify and speed up the treatment planning process and minimize the chance of error. Preloaded plans further minimize the risk of incorrect and hazardous optimization.

## Improvements

Numerous challenges have been encountered throughout the process of implementing and maintaining the HDR brachytherapy program in Nicaragua. Many of the challenges stem from finite technical and financial resources.

Limited technical resources often result in a delay or complete inability to provide quality patient care. In the setting of an HDR-source or software malfunction, outside expertise must travel to Nicaragua to troubleshoot. For example, when an old source needs to be exchanged, the center relies on the coordination of various parties to plan and approve the exchange. Significant delay in source exchange results in treatment of fewer patients, and sometimes the sending of patients to the next closest HDR center for treatment, which is in Costa Rica.

Additional technical constraints include a limited supply of brachytherapy applicators and dated technology. The center has three applicators that are all used throughout the day. After each insertion, the applicator is disinfected with a high-level disinfectant; there is no capacity for sterile processing of the instruments used. Currently, radiotherapy plans are digitized from 2-dimensional orthogonal images to reconstruct the applicator geometry. While this is a more time-efficient way of planning, the center’s staff have expressed interest in moving to 3-dimensional treatment planning if and when financial and technical resources are available.

Such limitations are largely attributable to limited financial resources. With increased financial autonomy, the center may be able to employ more staff (including physicians, residents, medical physicists, and radiation therapists), provide more treatment machines, and enhance current technologies – a struggle echoed throughout most LMIC.

## Discussion

The HDR program at El Centro Nacional de Radioterapia is a premier example of the effective implementation of an HDR brachytherapy program for the treatment of cervical cancer in a LMIC. As a systematic review of radiotherapy capacity in LMICs highlights, despite the dearth of publication on radiotherapy infrastructures in LMICs, such as Nicaragua, several Latin American countries are working to improve their RT delivery [4]. Additional publications identify areas of weakness within Latin America that may improve cancer care delivery throughout the region [5,6]; however, improvement of any kind is widely dependent upon the resources available within each country.

With the high burden of cervical cancer in Nicaragua, HDR brachytherapy remains at the cornerstone of quality cancer care. Reports of other LMICs with successful development of a brachytherapy treatment program include a recent report from Senegal as well as reports from the Botswana Oncology Global Outreach Collaborative partnership [7,8]. In particular, one recommendation from the report on Senegal’s brachytherapy program was to construct an HDR-dedicated room apart from an EBRT room. Nicaragua has successfully orchestrated a treatment program that moves patients between two treatment rooms; an HDR preparation room and an HDR treatment room. Both of these rooms are apart from the EBRT delivery rooms, so schedule coordination between the HDR suite and EBRT vault is not an issue. This process was made possible through a remote afterloading HDR unit as opposed to a unit located within the ^60^Co treatment vault.

Additionally, as has been discussed in previous reports and as we have reported here, a fixed-geometry applicator and library of standard plans help maximize treatment efficiency and accuracy [7]. Although transferring patients between the treatment preparation and treatment delivery rooms is not ideal, patients are kept on the same gurney throughout the treatment to minimize motion, and portable x-ray equipment in the treatment delivery room confirms adequate applicator placement prior to treatment delivery.

Amidst a high burden of cervical cancer incidence and mortality in Nicaragua, limited and finite resource availability remain an obstacle. Challenges in patient access, dated technology and software, and delays in equipment maintenance continue to limit the ability of oncologists in Nicaragua to provide curative treatment to women with cervical cancer. Our support for the HDR program in Nicaragua is ongoing, and we would like to highlight the valuable role of high-income countries in contributing to the resources and knowledge base in LMICs worldwide. Our relationship with El Centro Nacional de Radioterapia in Nicaragua provides a line of communication and collaboration in the hopes of continuing to help improve this already successful program.
